# Cysts of the Kidney

**Published:** 1903-01

**Authors:** 


### Cysts of the Kidney.

Kenneth A. J. Mackenzie, of Portland, Oregon, (*Med. Sentinel*, August, 1902,) says: simple or serous cysts are met with under various conditions. They are found in connection with the cirrhotic kidney, and are due to occlusion of the uriniferous tubules by encroachment of the neoplastic tissue. They are usually small, occasionally multiple, their growth is slow, and their fluid contains none of the urinary constituents. I have observed three cases in which there was nothing to suggest interstitial nephritis (three cases reported, complete cure in all three by nephrotomy, incision of the cyst, and drainage).

I have observed simple serous cysts in the young subjects in the post-mortem room. Serous cysts are more common in women than in men, in the proportion of twenty to three. Inasmuch as interstitial nephritis is more common in men than in women, the statement that the disease referred to is the constant concomitant of cystic kidney would seem to be fallacious. The diagnosis is difficult; they have been mistaken for omental, mesenteric, ovarian, pancreatic, hepatic and splenic cysts, also for hydatids, new growths and perinephritic effusions.

[illegible] POLYCYSTIC KIDNEY. CONGLOMERATE KIDNEY CYST.

The kidney is large in typical cases, and the whole organ appears converted into cysts. Cysts are found in the cortex, and medulla, and very frequently the cortex is entirely eclipsed by cysts. The cysts vary in size from a pea to an orange. The color of the cysts is often variegated, due to blood pigment alterations.

The specimen exhibited looked like a large bunch of fruit. Contents of the cysts are very often limpid, sometimes viscid and colloid, and sometimes they are solid and caseous. Both kidneys are generally involved, and sometimes the cysts involve the liver, spleen and other organs. Osler has recently called attention to the influence of heredity. The cyst walls are very thin, easily ruptured and are lined by thin tesselated epithelium; they seldom intercommunicate. The surviving tissue of the kidney shows atrophy and sclerosis.

Wilks and Moxon regard the condition as an advanced state of cystic disorder, found in granular kidney, and call attention to the identity of the circulatory disturbance in both diseases.

THEORIES EXPLAINING THE CONDITION.

I.

CONGENITAL INFLUENCES.

(*a*) Persistent germinal rudiments.

(*b*) Failure of union of the excretory canals with the convoluted tubules.

(*c*) Congenital feebleness of the walls of the tubules, resulting in dilatation, under the stress of the normal intrarenal pressure.

II.

Peritubular sclerosis, constriction of the tubules and backward dilatation of the Malpighian capsules, the disease being an essential part of cirrhosis of the kidney.

III.

Fetal inflammation of the papillæ, causing atresia and occlusion of the papillæ, and preventing communication between the uriniferous tubules and the calices of the kidney.

IV.

There is first local destruction of the tissue, followed by cystic or colloid degeneration.

V.

The cysts are neoplastic beginning in the epithelium of the tubules. Some authorities regard the disease as belonging to the adenomata.

The problem of etiology is to be studied in the light of the following questions:

(1) Is the development of the polycystic kidney common in the state known as granular kidney? (2) are the commonly accepted causes of granular kidney identical with those of polycystic kidney? (3) is congenital cystic kidney of the fetus or the new-born identical with the polycystic kidney of the adult? (4) is the age at which the disease develops the same as that of interstitial nephritis? (5) does the law of heredity apply in like manner to both diseases? (6) does the fact that cystic degeneration of the other organs, like the spleen and liver in the same individual at the same time, not suggest that it is a disease *per se*, independent of, and not associated with interstitial nephritis? (7) are the cardiovascular disturbances constant in polycystic kidney, and if so are they the same as those constantly associated with granular kidney? (8) is polycystic kidney associated with alcoholism, the gouty state or excesses in eating? (9) is there a tendency in polycystic kidney to anemia or dropsical effusions as in Bright's disease? (10) are the urinary modifications the same in the two diseases?

